# Exploring the effect and mechanism of fucoidan on liver cancer depending on network pharmacology and experimental verification

**DOI:** 10.3389/fchem.2025.1638785

**Published:** 2025-07-16

**Authors:** Xinghua Li, Chengyu Yang, Liwei Wang, Ihsan Ullah, Xinyue Liu, Chunqi Feng, Qi Liu

**Affiliations:** ^1^ College of Life Sciences, Shanxi University, Taiyuan, China; ^2^ Institute of Upper Gastrointestinal Tumour Prevention and Treatment, Shanxi Medical University, Changzhi, China; ^3^ Department of Gastrointestinal Surgery, Shanxi Province Cancer Hospital, Taiyuan, China; ^4^ School of Biomedical Sciences and Engineering, South China University of Technology, Guangzhou International Campus, Guangzhou, China; ^5^ School of Pharmacy, Shanxi Medical University, Taiyuan, China

**Keywords:** fucoidan, liver cancer, network pharmacology, experimental verification, Pi3k/AKt

## Abstract

**Objective:**

This research investigates the anti-liver cancer mechanisms of fucoidan by integrating network pharmacology analysis, molecular docking, and *in vitro* validation.

**Methods:**

Potential targets of fucoidan were first predicted using the SwissTargetPrediction platform. Subsequently, targets associated with liver cancer were identified through data extraction from three established databases: GeneCards, OMIM, and TTD, and the intersection between the targets of fucoidan and liver cancer was identified. A network integrating disease-associated and drug-target interactions was established by analyzing overlapping targets, and the core targets for fucoidan’s anti-liver cancer effect were identified through topological network analysis. Functional enrichment analyses, including Gene Ontology (GO) annotation and KEGG pathway analysis, were performed via the Hiplot platform. Molecular docking of the core targets with fucoidan was conducted using AutoDock to asses binding affinities. Finally, experimental validation was performed using real-time PCR and Western blotting to examine the effects of fucoidan on target proteins and signaling pathways.

**Results:**

A total of 69 common targets and 10 core targets were identified. These target genes were primarily involved in regulating biological processes such as cell apoptosis and proliferation, and were significantly associated with the PI3K/Akt and MAPK signaling pathways. Molecular docking demonstrated favorable binding affinities between fucoidan and the core target proteins. *In vitro* experiments revealed that fucoidan significantly inhibited the proliferation of HepG2 cells, downregulated the mRNA expression levels of AKT1, PI3K, PIK3R1, and PIK3CA, and reduced the protein expression of PI3K and Akt in the PI3K/Akt, indicating effective inhibition of the PI3K/Akt signaling pathway.

**Conclusion:**

Fucoidan exerts its anti-liver cancer effect primarily by downregulating mRNA expression levels of target genes including AKT1, PI3K, PIK3R1, PIK3CA and other targets, inhibition of PI3K/Akt signaling pathway activation, and suppressing HepG2 cell proliferation.

## 1 Introduction

Hepatocellular carcinoma (HCC) is one of the most common malignant tumors and represents a significant threat to human health. The International Agency for Research on Cancer’s 2024 report on worldwide cancer statistics states, In 2022, liver cancer incidence and death ranked sixth and third all over the world, respectively (Bray et al., 2024). In recent years, with the continuous improvement of socioeconomic development and changes in lifestyle and dietary patterns, the incidence and mortality rates of liver cancer have significantly increased. Alarmingly this trend is increasingly observed in younger populations (Zhang et al., 2022). The characteristics of liver cancer include insidious onset, subtle early clinical manifestations, diagnostic challenges, and poor prognosis. Most patients are diagnosed at advanced stages or after the onset of metastatic symptoms, with an around 12% 5-year rate of survival (Yin et al., 2023). Surgical resection remains the primary therapeutic approach, often complemented by postoperative radiotherapy, chemotherapy, and, in some cases, liver transplantation (Li D. et al., 2022). However, adverse effects such as disease recurrence, postoperative bleeding, and toxic reactions induced by chemotherapeutic agents (including gastrointestinal reactions, bleeding, renal toxicity, cardiac toxicity, hypertension, and hematological system damage) significantly impact patients’ health status and quality of life (Anwanwan et al., 2020).

Fucoidan is a sulfated polysaccharide derived from marine sources, primarily found in the cell wall matrix of various brown algae, such as kelp, Sargassum, and wakame (Jin et al., 2022; Sayuti et al., 2023). In many Asian countries, brown algae rich in fucoidan are widely utilized as both food and medicinal materials (Tung et al., 2022). Structurally, fucoidan is composed mainly of L-fucose and sulfate groups, with branched chained containing residues of glucose, galactose, xylose, mannose, and glucuronic acid (Haggag et al., 2023). It exhibits a broad spectrum of biological and pharmacological activities, including anticancer, antibacterial, antiviral, and anti-inflammatory, and anticoagulant effects (Dobrinčić et al., 2021). Its antitumor effects can be mediated through the regulation of the cancer cell cycle, inhibition of cell proliferation, induce apoptosis of cancer cells, or suppression of tumor angiogenesis, thereby blocking the spread and metastasis of cancer cells (Pan et al., 2019).

Recent studies suggest that fucoidan may play a crucial role in the therapy of liver cancer, capable of inhibiting tumor cell proliferation and metastasis through various mechanisms, thereby improving patient survival outcomes (Pan et al., 2019; Yang et al., 2013; Kawaguchi et al., 2015; Chen et al., 2024; Ma et al., 2021). For example, studies have shown that fucoidan can target integrin αVβ3 to inhibit liver cancer cells invasion and migration, thereby reducing the risk of transfer (Pan et al., 2019). Additionally, Fucoidan has been shown to induce G1 cell cycle arrest and promote apoptosis in hepatoma cell (Min et al., 2014). Despite its demonstrated anticancer potential, the precise molecular targets and mechanisms of fucoidan remain incompletely understood. To address this, integrative approaches combining bioinformatics, network pharmacology, and molecular docking have emerged as powerful tools to elucidate the molecular basis of fucoidan’s activity. These methods facilitate the identification of key genes and signaling pathways involved in liver cancer and reveal potential targets of fucoidan, providing a scientific rationale for its therapeutic application. This study aims to employ network pharmacology to identify core targets of fucoidan in liver cancer treatment, and to validate these targets through *in vitro* cell viability assays and molecular biology techniques, thereby establishing a theoretical foundation for future mechanistic investigations.

## 2 Materials and methods

### 2.1 Materials and reagents

Fucoidan was obtained from Qingdao Mingyue Hailin Fucoidan Biotechnology Co., Ltd. (Qingdao, China). The human HCC cell line HepG2 was purchased from Seven Biotech (Beijing, China), along with McCoy’s 5A medium. Fetal bovine serum and penicillin/streptomycin solution were sourced from CELL-BOX (Changsha, China) and Servicebio Technology Co., Ltd. (Wuhan, China), respectively. Cell viability was assessed using the CCK8 assay kit (Huiyucheng Biotechnology Co., Ltd., Wuhan, China). Isolation of total RNA by RNA extraction kit (Seven Biotech, Beijing, China), and mRNA expression levels were quantified via RT-qPCR using a two-step kit from the same supplier. An ultra-sensitive ECL chemiluminescent reagent kit (Abbkine Scientific Co., Ltd., Shanghai, China) was employed for protein detection. Primary antibodies specific for PI3K, phosphorylated PI3K (p-PI3K), AKT, and phosphorylated AKT (p-AKT) were supplied by Abmart Biomedical Co., Ltd. (Shanghai, China). The β-actin antibody was acquired from Proteintech Group, Inc. (Wuhan, China), and the secondary antibody, Goat Anti-Rabbit Mouse IgG-HRP, was also sourced from Abmart Biomedical Co., Ltd. (Shanghai, China).

### 2.2 Component-target-disease network analysis

The primary components of fucoidan were obtained through literature review, and their corresponding two-dimensional structures were retrieved from the PubChem database (https://pubchem.ncbi.nlm.nih.gov/) (Kim et al., 2016). Subsequently, the SwissTargetPrediction database (https://www.swisstargetprediction.ch/) (Gfeller et al., 2014) was utilized, setting the organism to “*Homo sapiens*,” and selecting results with a prediction possibility greater than 0 to identify the potential targets of fucoidan. Using the keyword “liver cancer,” retrieval of potential targets related to liver cancer from GeneCards database (https://www.genecards.org/) (Ben-Ari Fuchs et al., 2016), TTD database (http://db.idrblab.net/ttd/) (Chen et al., 2002), and OMIM database (https://www.ncbi.nlm.nih.gov/omim/) (Amberger and Hamosh, 2017). Subsequently, the targets obtained from the three databases were merged, and duplicates were removed to identify unique targets associated with liver cancer. The intersecting targets between fucoidan-related predictions and liver cancer-associated genes were determined to identify potential therapeutic targets. A compound-target interaction network was subsequently constructed using Cytoscape 3.8.0 (Doncheva et al., 2019).

### 2.3 Construction of the protein-protein interaction (PPI) network

The intersecting targets between fucoidan and liver cancer were imported into the STRING database (https://cn-db.org/) (Szklarczyk et al., 2019) for PPI analysis, WITH the organism set to “*H. sapiens*” was selected as the species, and the confidence level was modified to ≥0.7. Proteins lacking interaction data were excluded. The resulting PPI network was subsequently visualized and constructed using Cytoscape software (version 3.8.0). To identify hub genes potentially involved in the therapeutic effects of fucoidan on liver cancer, the CytoHubba plugin within Cytoscape was employed to analyze and rank key targets based on network topology.

### 2.4 Gene ontology (GO) and kyoto encyclopedia of genes and genomes (KEGG) pathway enrichment analysis

Hiplot (https://hiplot.com.cn/) was used to conduct Gene Ontology (GO) analysis and KEGG pathway enrichment analyses (Li J. et al., 2022). A p-value less than 0.05 indicates that the GO terms and KEGG pathways are statistically significant. The top 20 enrichment results were visualized. Additionally, a drug–target interaction network was constructed and subjected to further analysis.

### 2.5 Molecular docking

Molecular docking was performed on the core components and key targets obtained from network pharmacology analysis. Choose the appropriate target protein structure via the PDB database (https://www.rcsb.org/) (Protein Data Bank et al., 2019), defining the organism as “*H. sapiens*” and choosing structures with a resolution of less than 2.5 Å, while small molecules were included as reference benchmarks. The PubChem database was used to acquire the three-dimensional SDF files of fucoidan’s active components. Protein targets were dehydrated through the use of AutoDock software, adjust the size and position of the docking box, and optimize the conformations of the ligands and receptors. The results were output in “pdbqt” format. PyMOL 2.4.0 software was utilized to visualize the molecular docking results, analyzing docking affinity, hydrogen bonding, and bond lengths to reflect the stability of intermolecular interactions.

### 2.6 Cell activity determination by CCK8

HepG2 cells in the logarithmic growth phase were seeded into 96-well plates at an optimized density of 5,000 cells per well, with each well containing 100 µL. Fucoidan solutions were prepared in McCoy’s 5A medium across nine concentration gradients (0–6.4 mg/mL). Following a 24-h pre-culture at 37°C with 5% CO_2_, the medium was substituted with solutions containing fucoidan and incubated for another 24 h. Cell viability assessment was performed using CCK-8 assay under light-protected conditions: post-treatment medium removal, 10 µL CCK-8 reagent addition, thorough mixing, and 60min incubation. Absorbance was subsequently measured at 450 nm using microplate reader. Dose-dependent anti-proliferative effects were statistically analyzed and graphically represented through concentration-response curves.

### 2.7 Real-time qRT-PCR

Network pharmacology analysis identified AKT, PI3K, PI3KCA, and PI3KR1 as critical targets for investigating fucoidan’s influence on hepatocellular carcinoma cell proliferation. Total RNA was extracted from cells using the SevenFast Total RNA Extraction Kit, and RNA concentration was subsequently quantified. Was measured. From this RNA, complementary DNA (cDNA) was produced and then subjected to PCR amplification. The qRT-PCR was performed with the following thermal cycling conditions: initial denaturation at 95°C for 30 s, succeeded by 40 cycles of 95°C for 10 s, 60°C for 20 s, and 72°C for 20 s. The β-actin gene was used as an internal control, and the relative expression levels of the target genes were measured by applying the 2^−ΔΔCT^ formula. Seven biotech synthesized the primers, refer to Table 1 for the primer sequence.

**TABLE 1:** Primer sequence.

Gene	Sequence (5′-3′)
AKT1	5′-AGA​AGC​AGG​AGG​AGG​AGG​AG-3′
	5′-CCC​AGC​AGC​TTC​AGG​TAC​TC-3′
PI3K	5′-AAG​CAG​TGC​CTG​TAG​GAG​GA-3′
	5′-TGT​CGA​TGA​GCT​TTG​GTG​AG-3′
PIK3CA	5′-CAT​GCA​TTG​TTT​TGC​ACC​CC-3′
	5′-ATG​GAA​GAC​GGG​AGA​TTC​ACA​T-3′
PIK3R1	5′-TCT​ACC​CAG​TGT​CCA​AAT​ACC​AG-3′
	5′-TAA​ATG​CTT​CGA​TAG​CCG​TTC-3′
β-actin	5′-CAG​ATG​TGG​ATC​AGC​AAG​CAG​GA-3′
	5′-CGC​AAC​TAA​GTC​ATA​GTC​CGC​CTA-3′

### 2.8 Western blotting

Cells were cultured in two groups: a control group and a fucoidan-treated group. Cells from both groups were harvested upon reaching the logarithmic growth phase, and 400 μL of RIPA lysis buffer was used to lyse the cells. For a period of 30 min, the suspensions were kept chilled on ice. Afterward, the cell debris-containing lysates were transferred to centrifuge tubes and centrifuged at 12,000 revolutions per minute for a quarter of an hour at 4°C. The resulting supernatant was collected for protein concentration analysis. The isolated proteins were mixed with 5 × protein loading buffer in a 5:1 volume ratio and denatured at 100°C for 5 min. Each sample contained 20 μg of total protein for electrophoresis separation,and transfer onto PVDF membrane. Let the membrane be blocked with 5% skimmed milk for a duration of 2 hours. Following an overnight period at 4°C with the primary antibody, and then a 1-h incubation with secondary antibodies took place. For chemiluminescence detection, ECL reagent, consisting of an enhancer and stable peroxidase solution mixed in a 1:1 ratio, was applied to the PVDF membrane. Band intensity was measured using ImageJ software to evaluate relative protein expression levels across groups.

### 2.9 Statistical analysis

Statistical analysis of the experimental data was performed with the help of GraphPad Prism 8.0 and ImageJ software. The results are shown as the mean plus or minus the standard deviation (Mean ± SD). The analysis of differences between the two groups was performed using an independent samples t-test, while comparisons across several groups were conducted using a one-way analysis of variance (ANOVA). Statistical significance was assigned to P-values under 0.05 (*P* < 0.05).

## 3 Results

### 3.1 Analysis of the component-target-disease network

**FIGURE 1:**
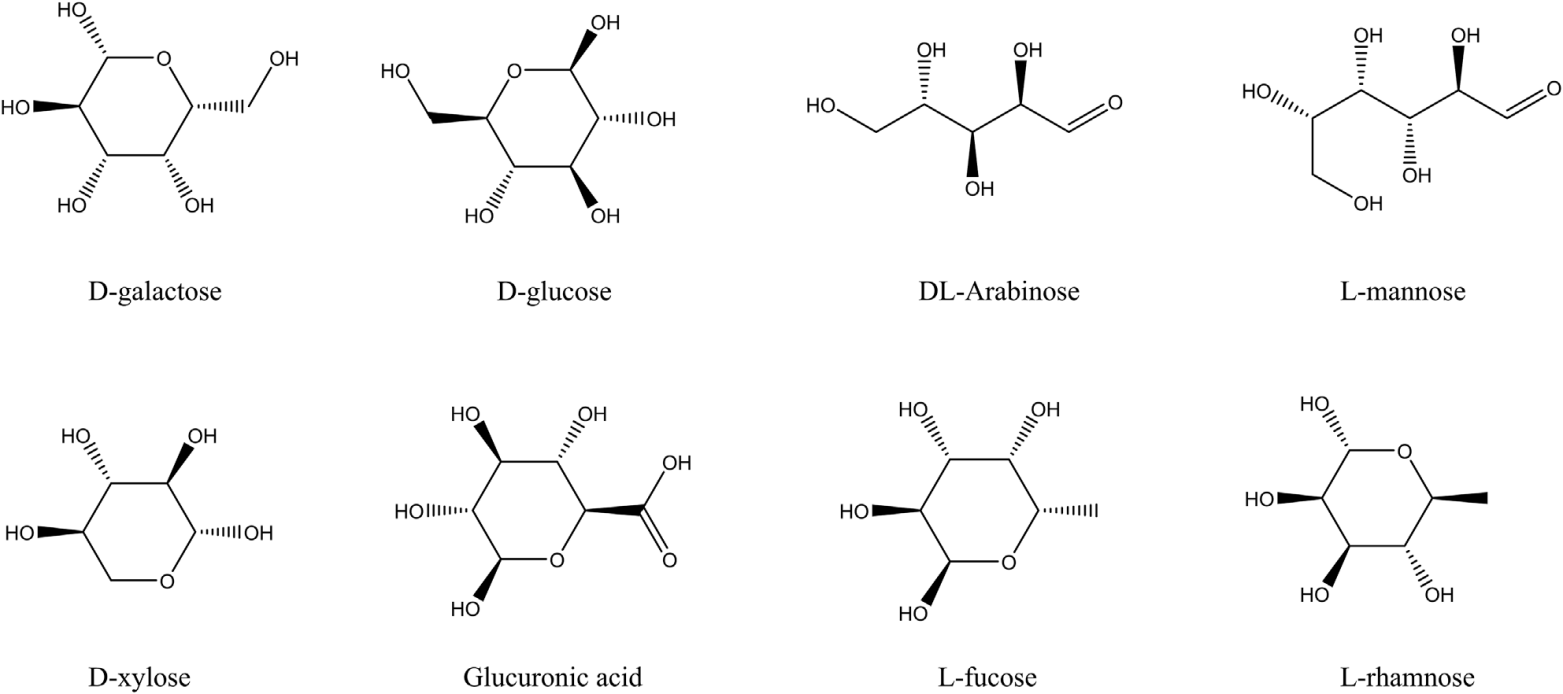
The main components of fucoidan.

**FIGURE 2:**
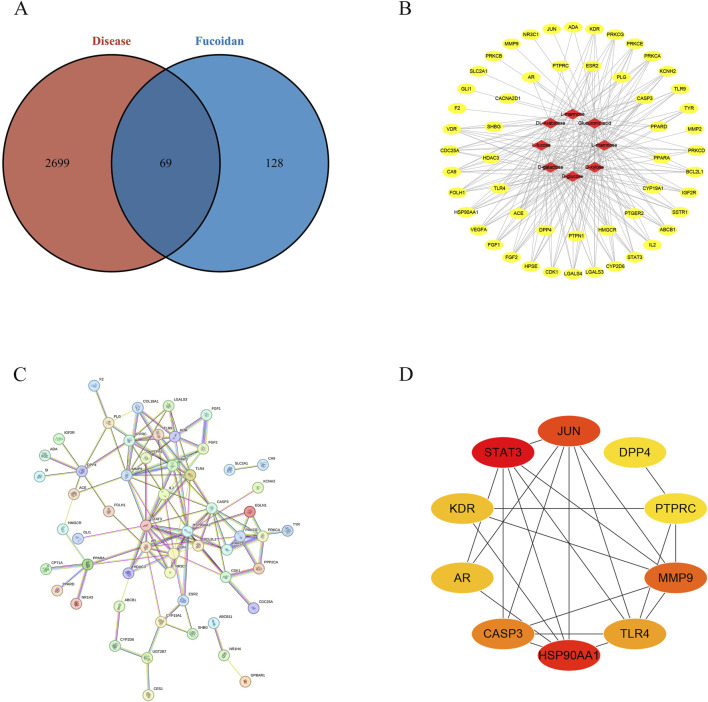
**(A)** The Venn diagram illustrating the shared targets between constituents and HCC-related pathways. **(B)** “Active ingredient-target” network diagram. **(C)** Diagram of the protein interaction network for potential targets. **(D)** The interaction networks of ten key targets.

The main components of fucoidan include D-galactose, L-rhamnose, L-mannose, L-fucose, D-glucose, D-xylose, DL-arabinose, and Glucuronic acid, as shown in Figure 1. These eight compounds were subjected to ADME screening, and their potential targets were identified by analyzing SMILES structures with the Swiss Target Prediction. A total of 197 compound-related genes were identified. Meanwhile, 2768 genes linked to liver cancerwere retrieved from the database. The analysis using a Venn diagram found 69 targets that overlapped between the 197 fucoidan-related genes and the 2768 liver cancer-associated genes, as shown in Figure 2A. Using Cytoscape 3.8.0, a network associating components with targets was created, integrating eight active compounds and 69 overlapping genes to highlight their roles in fucoidan’s therapeutic effects on liver cancer, as shown in Figure 2B.

### 3.2 Construction of the protein-protein interaction (PPI)

A protein-protein interaction network was constructed by inputting the 69 identified targets into the STRING database, using a confidence threshold of 0.700, focusing on *Homo sapiens*, as shown in Figure 2C. The resulting interaction data were imported into Cytoscape 3.8.0 for topological analysis, using the Cytohubba tool. According to degree centrality, HSP90AA1, JUN, PTPRC, MMP9, DPP4, TLR4, STAT3, AR, CASP3, and KDR was listed in the top ten targets, as shown in Figure 2D.

### 3.3 Analyses using gene ontology (GO) and the kyoto encyclopedia of genes and genomes (KEGG)

**FIGURE 3:**
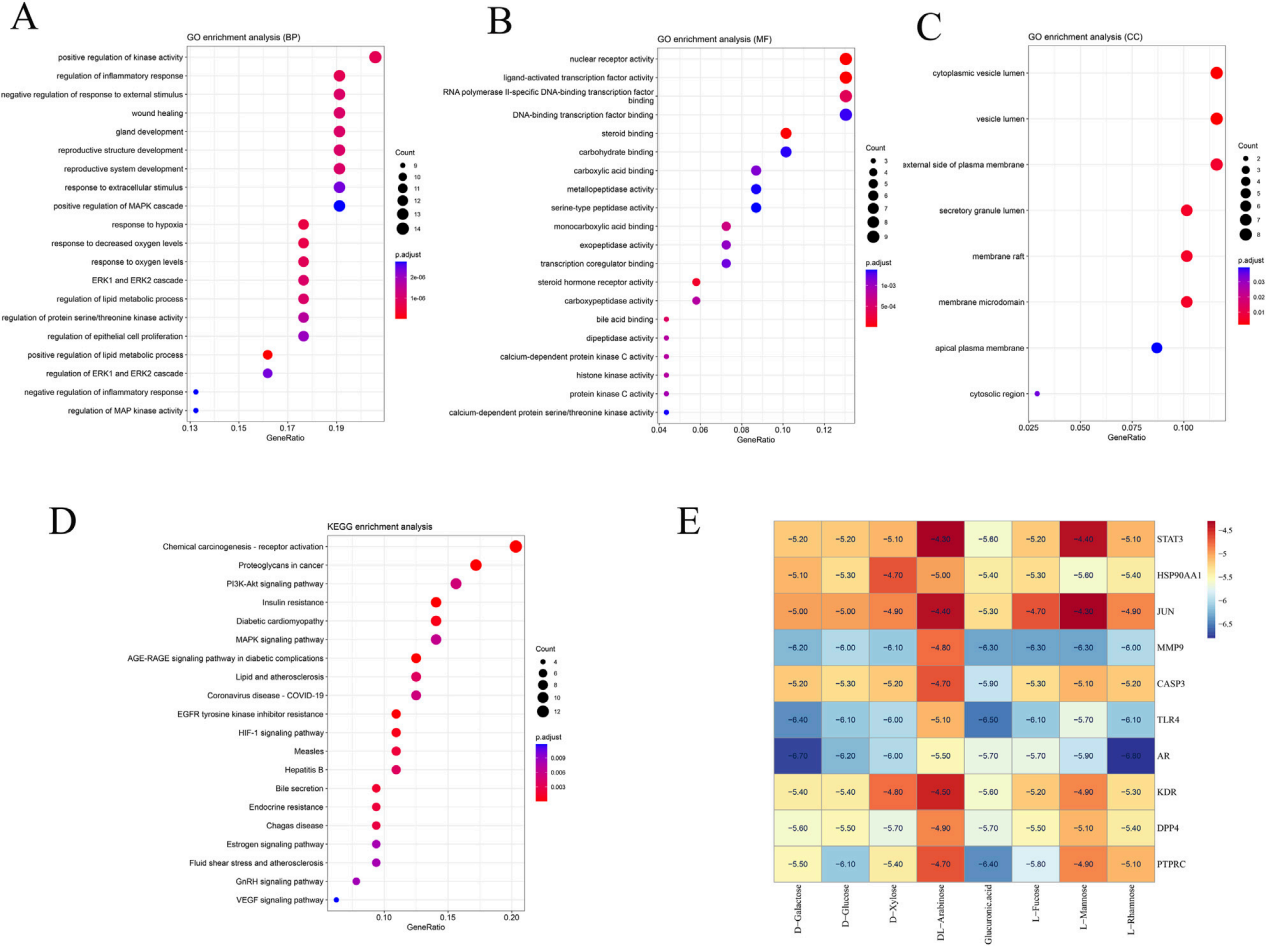
Enrichment analysis of potential targets derived from the primary active ingredients of Fucoidan. **(A)** Gene Ontology terms related to biological processes. **(B)** Gene Ontology terms associated with molecular function. **(C)** Gene Ontology terms related to cellular components. **(D)** KEGG pathways. **(E)** Molecular docking heat map.

To explore the potential mechanisms of action in liver cancer treatment, Go, and KEGG analysis were performed on the 69 identified targets. The top 20 GO terms were categorized into biological process (BP), cellular component (CC), and molecular function (MF), revealing significant associations with the cytoplasmic vesicle lumen, membrane raft, and vesicle lumen. These targets contributed to the regulation of molecular functions, including the activity of transcription factors activated by ligands, binding of steroids, serine-type peptidase activity, and growth factor receptor binding, as shown in Figures 3A–C. The 69 overlapping genes were significantly enriched (p < 0.05) in 20 signaling pathways, according to KEGG analysis, including Chemical carcinogenesis - receptor activation, Proteoglycans in cancer, PI3K-Akt signaling pathway, and MAPK signaling pathway, as shown in Figure 3D.

### 3.4 Molecular docking

**FIGURE 4:**
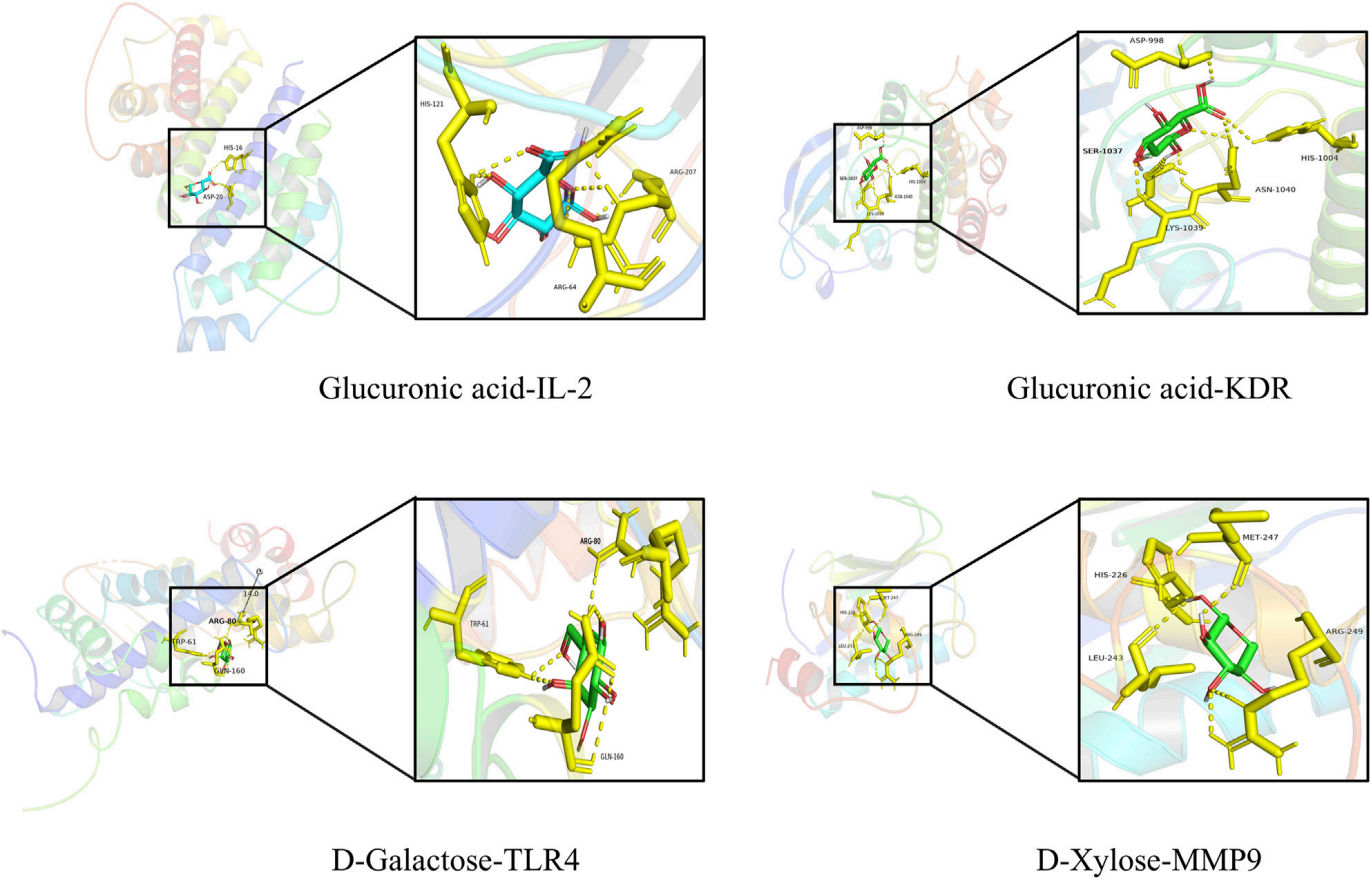
Schematic representation of molecular docking.

In order to additionally confirm the precision of network pharmacology, the binding affinities of major active compounds with hub proteins were assessed through molecular docking analysis using AutoDock Vina software. Each of the eight compounds exhibiting activity had binding affinities with the ten hub proteins that were under −4 kcal/mol, revealing good binding capability. A heatmap was generated to illustrate the strong binding affinities of fucoidan’s core targets and active components, as shown in Figure 3E. Some molecular docking results were visualized using PyMOL 2.4.0, as shown in Figure 4.

### 3.5 Cell activity determination by CCK8

**FIGURE 5:**
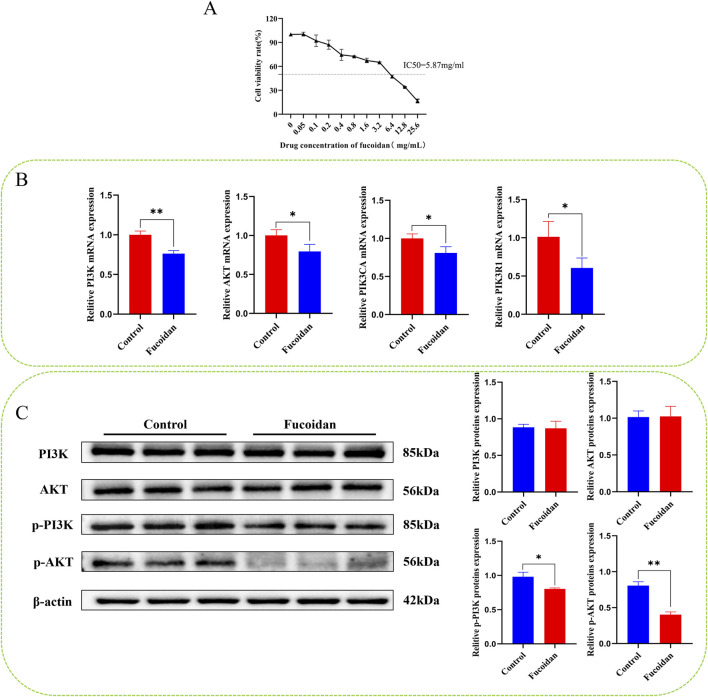
**(A)** The impact of fucoidan on the viability of HepG2 cells. **(B)** Impact of fucoidan on the mRNA expression levels of PI3K, AKT, PIK3CA, and PIK3R1 (*p < 0.05, **p < 0.01). **(C)** Impact of fucoidan on the protein expression levels of pPI3K and p-AKT (*p < 0.05, **p < 0.01).

The cytotoxicity of fucoidan on HepG2 cells exhibited a clear concentration-dependent pattern. As determined by the CCK8 assay, the cell inhibition rate progressively increased with rising concentrations of fucoidan. Based on the dose-response curve, the IC50 value of fucoidan for HepG2 cells was computed to be around 5.87 mg/mL. These findings validate the cytotoxic impact of fucoidan on HepG2 cells as well as support the selection of the IC50 concentration for future experiments. Statistically, the observed differences were highly significant (*p < 0.05, **p < 0.01), as demonstrated in Figure 5A.

### 3.6 Real-time qRT-PCR

Real-time PCR analysis revealed that mRNA levels of AKT1, PI3K, PIK3R1, and PIK3CA were significantly reduced in the fucoidan-treated group compared to the control group (*p < 0.05, **p < 0.01), as demonstrated in Figure 5B.

### 3.7 Western blotting

Western blot analysis showed there were no notable differences in the protein levels of PI3K and AKT1 between the group that was treated with fucoidan and the control group. However, the levels of p-PI3K and p-AKT1 were significantly reduced (*p < 0.05, **p < 0.01), as demonstrated in Figure 5C.

## 4 Discussion

Fucoidan is a natural sulfated polysaccharide, which mainly exists in brown algae. (Richards et al., 2020). It exhibits broad pharmacological effects and low toxicity, making it a promising source of anticancer agents. Current cancer treatment methods typically include chemotherapy, radiotherapy, and immunotherapy (Klika et al., 2024; Makarem et al., 2025); however, these approaches may be limited by adverse side effects and mechanisms of drug resistance. Recent studies have highlighted the potential of fucoidan as an adjunctive therapeutic agent, demonstrating its ability to inhibit tumor growth, induce apoptosis in cancer cells, and modulate immune responses. These findings underscore the significance of fucoidan in enhancing the efficacy of traditional therapies while minimizing toxicity.

This research used network pharmacology to discover possible targets and signaling pathways linked to fucoidan in combating liver cancer, resulting in 197 targets associated with fucoidan and 2,768 targets related to liver cancer, with 69 overlapping targets. A PPI network of the intersecting genes between fucoidan and liver cancer was established, revealing that targets such as HSP90AA1, JUN, PTPRC, MMP9, DPP4, TLR4, STAT3, AR, CASP3, and KDR ranked significantly high, providing important clues for further exploration of the potential mechanisms by which fucoidan regulates liver cancer. HSP90AA1 is a molecular chaperone protein that promotes tumor cell survival by stabilizing a variety of oncogenic proteins, such as kinases, transcription factors, and hormone receptors. It is overexpressed in a variety of cancers and is associated with tumor progression, metastasis and drug resistance. Inhibition of HSP90AA1 expression can reduce tumor cell proliferation (Rashmi et al., 2017). STAT3 is a transcription factor that is involved in a variety of biological processes such as cell proliferation and differentiation and angiogenesis, but is overactivated in most cancer cells. As a transcription factor, STAT3 regulates a series of genes associated with biological processes such as cancer cell hyperproliferation, invasion and metastasis, and immune evasion (Song et al., 2011). Growing evidence indicates that inhibiting STAT3 activity in cancer cells can suppress tumor growth and increase chemotherapy sensitivity in HCC cells (Chen et al., 2017). CASP3 is an executive protein for tumor cell apoptosis, and activation of CASP3 can promote apoptosis of liver cancer and inhibit tumor growth (Li et al., 2013). Elevated levels of MMPs, especially MMP-9, are considered an important factor in hepatocarcinogenesis and a promoter of tumor invasion and angiogenesis (Roomi et al., 2019). These core targets may be closely related to fucoidan’s anti-liver cancer, inhibition of liver cancer cell proliferation and promotion of apoptosis in liver cancer cells. The molecular docking results further showed that the core compounds of have high binding activities with these core proteins.

Additionally, Analysis of target functional enrichment analysis indicated that fucoidan’s anti-liver cancer activity is primarily associated with the PI3K/Akt signaling pathway. This pathway a pivotal role in the progression of tumors and is intimately linked to the control of various biological processes associated with tumors, making it a focal point for developing new therapeutic targets for liver cancer. Activation of the PI3K/Akt pathway promotes the migration, invasion, and proliferation of liver cancer cells (Min et al., 2014), while inhibition of this pathway can suppress the proliferation and metastasis of liver cancer cells (Richards et al., 2020). It has been found that cancers with activated PI3K/Akt signaling become more aggressive, and in clinical liver cancer patients, activation of the AKT pathway is an important indicator of risk for an early relapse and an unfavorable outcome (Tang et al., 2022). Once activated, Akt initiates downstream signaling cascades, including phosphorylation of mTOR and activation of NF-κB, thereby promoting tumor growth, enhancing chemoresistance, and inhibiting apoptosis (Tang et al., 2022; Ha et al., 2021; Buontempo et al., 2011). Therefore, this study selected PI3K and AKT as key factors of the pathway of PI3K/Akt signaling for conducting cellular experiments *in vitro*. Results from Western blotting and RT-qPCR demonstrated that fucoidan has the potential to diminish the expression levels of PI3K/Akt proteins in liver cancer cells. This effect has the potential to cause apoptosis in liver cancer cells through the downregulation of the PI3K/Akt signaling pathway, which matches the conclusions drawn from the network pharmacology analysis.

The results of this study provide a foundation for further exploration of fucoidan as a potential therapeutic agent for liver cancer. Given its ability to modulate critical oncogenic pathways and induce apoptosis in cancer cells, fucoidan may serve as an adjunctive therapy to enhance the efficacy of conventional treatments such as chemotherapy or targeted therapy. Notably, fucoidan’s natural origin and reported low toxicity in previous studies suggest its potential as a safe and well-tolerated option for long-term administration in cancer patients. Future clinical studies should focus on evaluating the pharmacokinetics, optimal dosing, and synergistic effects of fucoidan with existing liver cancer therapies. Additionally, the identified hub targets could be further validated as biomarkers for patient stratification or therapeutic response monitoring. This study not only advances the understanding of fucoidan’s anti-liver cancer mechanisms but also highlights its translational potential in clinical oncology. Further preclinical and clinical investigations are warranted to fully exploit its therapeutic benefits.

While our study primarily focused on elucidating the network pharmacology-driven mechanisms of fucoidan against liver cancer, we acknowledge that its well-documented immunomodulatory effects were not experimentally validated in this work. Given that fucoidan has been reported to modulate immune responses—such as enhancing natural killer (NK) cell activity and macrophage polarization—these properties could synergistically contribute to its anti-tumor efficacy in liver cancer. The absence of direct immunological data represents a limitation of the current study, and future investigations should explore immune-related pathways (e.g., PD-1/PD-L1 axis or cytokine profiles) to provide a more comprehensive understanding of fucoidan’s therapeutic potential. Such studies could bridge the gap between its pharmacological targets and immune-mediated anti-cancer effects.

This investigation points out the possibility of fucoidan in liver cancer treatment. As a promising anticancer active component, it may open new possibilities for liver cancer therapy. Nonetheless, it's crucial to recognize that fucoidan, as a plant polysaccharide, still faces challenges such as low bioavailability, lack of safety analysis, and inconsistencies in therapeutic doses between *in vitro* and *in vivo* studies. Addressing these issues will be a key focus of our future research.

## 5 Conclusion

In summary, this study integrated network pharmacology and molecular docking analysis to identify potential targets, and elucidate the underline mechanism of fucoidan in the treatment of liver cancerUltimately, *in vitro* cellular experiments confirmed its suppressive impact on the viability of liver cancer cells and its ability to induce apoptosis in these cells, suggesting that the anticancer mechanism of fucoidan could possibly be associated with the downregulation of the pathway of PI3K/Akt signaling. Future investigations involving *in vivo* models are warranted to further validate this mechanism. Overall, this study offers a methodological framework for exploring the multi-target and multi-pathway actions of traditional Chinese medicine in liver cancer therapy and provides a valuable reference for advancing the clinical application of medicinal and dietary polysaccharides in oncology.

## Data Availability

The original contributions presented in the study are included in the article/Supplementary Material; further inquiries can be directed to the corresponding author.
